# Supplementary material for: Comparative Evaluation of GS-441524, Teriflunomide, Ruxolitinib, Molnupiravir, Ritonavir, and Nirmatrelvir for In Vitro Antiviral Activity against Feline Infectious Peritonitis Virus
Source: Vet Sci. 2023 Aug 9;10(8):513. doi: 10.3390/vetsci10080513 (PMC10459838; doi:10.3390/vetsci10080513)
Supplement: Supplementary file 1 [file vetsci-10-00513-s001.zip › Supplementary Figure S1.pdf]

A B

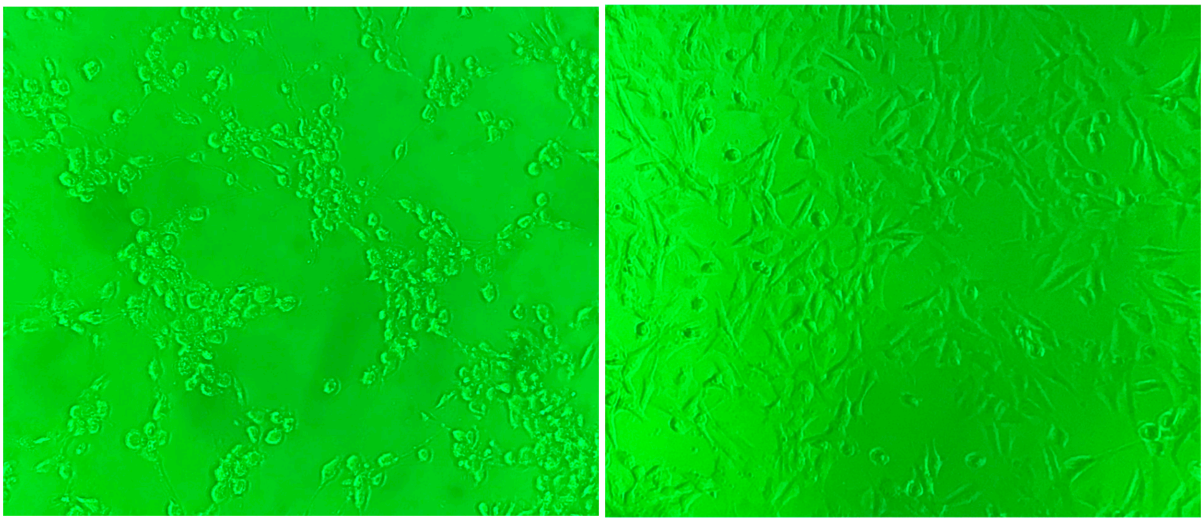

Supplementary Figure S1. Visualization of CPE in drug treated CRFK cells. The visual inspection of the antiviral drug-treated wells just before applying the fluorescence dye and plate readings revealed the differences in cell morphology (CPE) between the treated (**A**) and untreated (**B**) CRFK cells in cell cytotoxicity assay. The inconsistency between the visual assessment of drug-treated wells and the fluorescence assay led to the possibility that an overall decrease in the cell number in the drug-treated wells resulted in degradation and loss of nucleic acid, thus being unavailable for fluorescence binding and detection in the CellTox assay. As a result, when the cell viability is near to 0% at a high drug concentration, the corresponding concentration's percent cytotoxicity is much lower than 100%.
