# Supplementary material for: Comparative Evaluation of GS-441524, Teriflunomide, Ruxolitinib, Molnupiravir, Ritonavir, and Nirmatrelvir for In Vitro Antiviral Activity against Feline Infectious Peritonitis Virus
Source: Vet Sci. 2023 Aug 9;10(8):513. doi: 10.3390/vetsci10080513 (PMC10459838; doi:10.3390/vetsci10080513)
Supplement: Supplementary file 1 [file vetsci-10-00513-s001.zip › Table S1.pdf]

Table S1. Percent inhibition response by six drugs (serial 1:10 dilutions) against FIPV in CRFK cells

Drug (µM)	GS441524			Teriflunomide			Ruxolitinib			Molnupiravir			Ritonavir			Nirmatrelvir		
	**Copy number**	**% inhibition**	**CPE**	**Copy number**	**% inhibition**	**CPE**	**Copy number**	**% inhibition**	**CPE**	**Copy number**	**% inhibition**	**CPE**	**Copy number**	**% inhibition**	**CPE**	**Copy number**	**% inhibition**	**CPE**
**0.00**	4.36×10^{6}	0.00	Yes	4.36×10^{6}	0.00	Yes	4.36×10^{6}	0.00	Yes	4.36×10^{6}	0.00	Yes	8.06×10^{6}	0.00	Yes	8.06×10^{6}	0.00	Yes
**0.05**	2.52×10^{6}	42.18	Yes	4.32×10^{6}	0.92	Yes	7.00×10^{6}	0.00	Yes	2.18×10^{6}	49.88	Yes	6.82×10^{6}	15.40	Yes	8.04×10^{6}	0.29	Yes
**0.50**	6.62×10^{5}	84.82	Yes	3.65×10^{6}	16.37	Yes	1.47×10^{6}	66.37	Yes	2.47×10^{6}	43.37	Yes	8.73×10^{6}	0.00	Yes	8.79×10^{6}	0.00	Yes
**5.00**	2.67×10^{5}	93.89	No	2.72×10^{6}	37.67	Yes	3.38×10^{6}	22.54	Yes	1.52×10^{6}	65.16	Yes	6.91×10^{6}	14.28	Yes	1.58×10^{6}	80.38	No
**50.00**	1.34×10^{4}	99.69	No	7.62×10^{5}	82.52	Yes	3.62×10^{6}	16.92	Yes	1.14×10^{6}	73.83	Yes	2.81×10^{4}	99.65	Yes	3.18×10^{4}	99.61	No
**500.00**	1.37×10^{4}	99.69	Yes	3.27×10^{4}	99.25	Yes	4.51×10^{4}	98.97	Yes	1.71×10^{5}	96.07	Yes	3.38×10^{4}	99.58	Yes	2.38×0^{4}	99.70	Yes
