# Supplementary material for: Comparative Evaluation of GS-441524, Teriflunomide, Ruxolitinib, Molnupiravir, Ritonavir, and Nirmatrelvir for In Vitro Antiviral Activity against Feline Infectious Peritonitis Virus
Source: Vet Sci. 2023 Aug 9;10(8):513. doi: 10.3390/vetsci10080513 (PMC10459838; doi:10.3390/vetsci10080513)
Supplement: Supplementary file 1 [file vetsci-10-00513-s001.zip › Table S2.pdf]

Table S2. Percent inhibition response by six drugs (1:2 dilutions) against FIPV in CRFK cells

Drug (µM)	GS441524			Teriflunomide			Ruxolitinib			Molnupiravir			Ritonavir			Nirmatrelvir		
	Copy number	% inhibition	CPE	Copy number	% inhibition	CPE	Copy number	% inhibition	CPE	Copy number	% inhibition	CPE	Copy number	% inhibition	CPE	Copy number	% inhibition	CPE
**0.00**	3.36×10^{6}	0.00	Yes	3.36×10^{6}	0.00	Yes	3.36×10^{6}	0.00	Yes	3.36×10^{6}	0.00	Yes	3.36×10^{6}	0.00	Yes	3.36×10^{6}	0.00	Yes
**0.98**	2.60×10^{6}	22.50	Yes	2.27×10^{6}	32.27	Yes	3.48×10^{6}	0.00	Yes	3.47×10^{6})	0.00	Yes	4.33×10^{6}	0.00	Yes	2.93×10^{6}	12.74	Yes
**1.95**	1.23×10^{6}	63.34	Yes	1.66×10^{6}	50.67	Yes	2.13×10^{6}	36.66	Yes	2.63×10^{6}	21.61	Yes	4.60×10^{6}	0.00	Yes	2.02×10^{6}	39.94	Yes
**3.90**	4.08×10^{5}	87.84	Yes	2.02×10^{6}	39.87	Yes	2.58×10^{6}	23.25	Yes	2.07×10^{6}	38.30	Yes	3.20×10^{6}	4.62	Yes	1.05×10^{6}	68.83	Yes
**7.80**	5.87×10^{4}	98.25	No	1.32×10^{6}	60.58	Yes	4.52×10^{6}	0.00	Yes	2.06×10^{6}	38.52	Yes	2.90×10^{6}	13.64	Yes	5.62×10^{4}	98.33	No
**15.65**	3.93×10^{4}	98.83	No	1.50×10^{6}	55.29	Yes	6.17×10^{6}	0.00	Yes	1.01×10^{6}	69.84	Yes	2.57×10^{6}	23.55	Yes	3.72×10^{4}	98.89	No
**31.25**	3.92×10^{4}	98.83	No	1.17×10^{6}	65.14	Yes	4.55×10^{6}	0.00	Yes	7.89×10^{5}	76.48	Yes	7.39×10^{5}	77.99	Yes	2.78×10^{4}	99.17	No
**62.50**	4.13×10^{4}	98.77	No	1.29×10^{6}	61.70	Yes	1.48×10^{6}	56.04	Yes	5.83×10^{5}	82.63	Yes	5.61×10^{4}	98.33	Yes	3.70×10^{4}	98.90	No
**125**	3.34×10^{4}	99.00	No	5.12×10^{5}	84.73	Yes	1.99×10^{6}	40.83	Yes	1.89×10^{5}	94.38	Yes	5.60×10^{4}	98.33	HT	3.24×10^{4}	99.04	No
**250**	6.32×10^{4}	98.12	Yes	3.33×10^{5}	90.09	Yes	2.40×10^{6}	28.61	Yes	6.08×10^{4}	98.19	Yes	5.61×10^{4}	98.33	HT	3.38×10^{4}	98.99	Yes
